# Drug Eruptions: Their Nature and Varieties

**Published:** 1911-01-21

**Authors:** J. H. Stowers

**Affiliations:** Vice-President of the Section of Dermatology, Royal Society of Medicine; late Physician to the Department for Diseases of the Skin, N.W. London Hospital; formerly President of the Dermatological Society of Great Britain and Ireland.


### HOSPITAL CLINICS.

### DRUG ERUPTIONS: THEIR NATURE AND VARIETIES—III.

By J. H. STOWERS, M.D., Vice-President of the Section of Dermatology, Royal Society of Medicine; late Physician to the Department for Diseases of the Skin, N.W. London Hospital; formerly President of the Dermatological Society of Great Britain and Ireland.

(Lecture at the Polyclinic, Chenies Street, W.C.)

To recapitulate, the active principles of drugs exercise their effects in the production of cutaneous eruptions, according to Fox: (1) Upon the vaso-motor or trophic nerves (central or peripheral), (2) upon special tissues, as skin, nerve structure, etc.; (3) upon special glands or appendages irritated during the process of elimination; (4) upon natural function by the production of excessive action; (5) upon the blood-vessels or their contents. In 1894 I had a coloured drawing made showing the eruption which occurred over the entire body and extremities of a male patient who was admitted into hospital after swallowing a quantity of oxalic acid with suicidal intent. The man was dangerously ill for several weeks, but eventually recovered, the severe vomiting immediately produced by the drug having saved his life. The eruption was papulo-erythematous and very acute, the appearance being somewhat similar to that caused in some instances by chloral hydrate, but much more severe in degree—that is to say, papular elevations upon an erythematous base. It is impossible now even to enumerate the drugs included in this group—exceeding a hundred in number—or the many plants which are productive of marked effects when applied to the skin, such as rhus toxicodendron (or poison ivy), rhus venenata, rhus vernicifera (Japan), primula obconica, chrysanthemum, various species of cypripedium (*C. spectabile*, *C. pubescens*, and *C. parviflorum*), producing great irritation on account of the glandular hairs when touched emitting an irritating substance, and others. Suffice it to say that the effects may be very severe, even to the extent of a general dermatitis, including acute ophthalmia, and upon the skin vesicles, pustules, erysipelatous reddening and œdema, with acute pain and sometimes disturbance of the kidney secretion.

In the case of the rhus vernicifera the stem and under surfaces of the leaves are covered with hairs containing a milky juice, and which, when dried, constitutes the dark-coloured varnish so much in use. It is to be noted that unexposed parts of the body frequently become secondarily infected by the hands.

Handling of hyacinth bulbs also irritates some people. Dr. Freeman, of Reading, has pointed out that a minute parasite is an important factor besides the raphides, or crystals of acicular form, occurring in plant cells.

I have seen also cases of dermatitis of both hands and forearms due to contact with the young shoots of the cow-parsnip (*Heracleum giganteum*), and others, more common, due to the plant angelica, which is used largely for the purpose of preserving, sweetmeats, etc.

### ACTION OF ARSENIC.

The very important influence of arsenic upon the skin calls for a special reference. With the increase of knowledge as to causation of disease many drugs which were formerly used in wholesale quantities are now happily prescribed in moderate doses only, and those in the particular stages of the ailment for which they are suitable. The time has gone by when arsenic was practically considered to be the only appropriate drug for internal use in dermatological practice. That its efficacy is great under special conditions we are all agreed, but that is, generally speaking, in the later stages of some ailments, and certainly not in the early and acute inflammatory conditions in which itching and pain are the prominent symptoms. For example, we should not now think of using it in acute lichen planus, an affection characterised by intense itching and the presence of those peculiar red, raised, shiny, flat and angular papules which spread by aggregation, and, when advanced, often produce hypertrophied plaques extending more or less over the whole surface of the limbs and trunk. In the late stage, however, when the inflammatory concomitants are absent, it may be, and frequently is, of great service.

Arsenical herpes is really a neuritis produced by the specific influence of the drug. The remarkable and extensive pigmentations of the skin, with or without keratosis, consequent upon its long-continued administration, was for many years overlooked, but now it is comparatively rarely seen. Illustrations have been published of cancerous-like looking ulcerations secondary to chronic keratoses, which have been described by the name of "arsenic cancer."

It would be impossible in this connection to overlook the historical investigations of Drs. Brooke and Leslie Roberts published in 1901 relative to the action of arsenic on the skin as observed in the epidemic of "arsenical beer poisoning," and of Dr. E. S. Reynolds' publication entitled "An Account of the Epidemic Outbreak of Arsenical Poisoning occurring to Beer-drinkers in the North of England and Midland Counties in 1900," for since the great epidemic of 1828 in Paris no such opportunity has occurred of studying the wholesale ravages produced by the toxic effects of this powerful drug. Brooke and Roberts state that "the most striking feature, about which the chief complaint was made, was that the hands and feet were swollen and hot, and so painful that it was impossible for the patients to continue their work. The palms and soles were red and often tender enough to make walking or the handling of objects unbearable, and these symptoms, in union with the numbness, tingling, formication, and hyper-

æsthesia which were present in greater or less degree, united to make up something closely akin to the well-known picture of erythromelalgia of Weir-Mitchell.

The lesions were not by any means confined to the palms and soles; the redness spread between the fingers on to the wrists and backs of hands and feet. The faces of a number of patients were swollen, flushed, and cyanosed. Some exhibited on the body and limbs rashes of a scarlatiniform or morbilliform kind. "By far the most striking of the cases were those in which the limbs, and often the body, were covered with red, bluish, or livid blotches of varying sizes and shapes." Desquamation after the manner of exfoliative dermatitis sometimes followed. The chief manifestations to which attention should be drawn as a consequence of the excessive administration of arsenic are herpetic and pemphigoid eruptions, excessive pigmentation (melanosis), hyperkeratosis, and desquamation, and arsenical warts which may grow and harden with pressure until they assume remarkable size and shape.

In relation to eruptions connected with the use of enemata they are generally well known. A case has been recorded in which urticaria invariably followed the passage of a sound into the uterus. An urticarial rash has been known to follow tapping for pleuritic effusion. Again, an eruption of similar kind has frequently followed paracentesis abdominis for hydatid cysts, and even spontaneous rupture of the same. The rash usually makes its appearance shortly after the puncture, and is generally accompanied by elevation of body temperature and abdominal pain, and occasionally giddiness and vomiting. It may also appear in the typical form of intensely itchy urticaria, or assume a diffuse erythema or a limited capillary injection of the surface in the shape of irregular scattered blotches. Various explanations have been put forward, such as the quality of the fluid secreted, a toxin in fact, and reflex influences through the nervous system.

Those who are in the habit of using the new vaccine treatment of disease are not infrequently encountering eruptions upon the skin of a similar kind. It has been noticed that the smaller the volume of serum in which a given number of units is injected, the more prompt is the effect and the less likelihood is there of cutaneous eruptions arising. This applies especially to anti-diphtheria serums.

It has been suggested by Netter that as a means of reducing this occurrence gramme doses of chloride of calcium may be introduced subcutaneously on the day of the injection, and Sir Almroth Wright suggests as a means of obviating such symptoms that adults should receive 30 grains of calcium chloride or calcium lactate daily from the sixth to the tenth day after the injection of the serum. Green relates a case in which urticaria and œdema followed the use of anti-diphtheritic serum and quickly yielded to 20-grain doses of calcium chloride given every two hours. Moreover, it has been stated by Roehr, of Chicago, that urticaria, œdema, and arthritis, following the use of diphtheria antitoxin, are effectually relieved by the administration of acetate of potassium, which aids elimination of the serum. It may be accepted that eruptions can arise from all antitoxic and antibacterial agents, as well as normal organic substances such as thyroid extract, if injected into the system through the circulation. The importance of recognising those which occasionally follow the use of calf lymph in vaccination and re-vaccination is necessarily very great, especially at this time, when the anti-vaccination craze amongst the ignorant and prejudiced is so rife. The explanation is probable, as may happen concurrently with small-pox, typhoid, and other infectious diseases, that bacterial toxins existing in the body are liberated by the organisms. The so-called ptomaines—due to decomposition of the elements of food—lead to cutaneous manifestations in some respects very similar to those produced by drugs. It is common experience now that sewer-gas poisoning is frequently followed by a remarkable erythematous eruption of general character. Crocker reported a case of this kind in which, so far as the eruption was concerned, it was impossible to distinguish between scarlatina and the scarlatiniform eruption. The suggestion has also been made that lupus erythematosus may depend upon toxins absorbed from deposits of tubercular or other morbid material existing within the body. Trousseau long since laid stress upon the "excretory irritation" of sweat in certain constitutional diseases in addition to drug eruptions proper.

I should have mentioned in connection with ptomaine poisoning that the borates and salicylates, whether administered for medicinal purposes or as so-called food preservatives, are frequently followed by eruptions upon the skin. In addition to the older remedies, such as bismuth, opium, morphia, and quinine, the effects of which are so well known, among the more recent therapeutic agents now in use I may include the numerous synthetical preparations, and especially antipyrin, anti-febrin, synphenyl-acetamide, phenacetin, exalgin, europhen, and their allies, as responsible for cutaneous manifestations of the erythematous and exudative type, as well as the so-called industrial poisonings which are so numerous. Instances have also been recorded of acute dermatitis, more or less general in character, from the local application of ichthyol, metol, thiosinamine, formalin, phenyl-hydrazine-hydrochloride, orthoform, possessing local analgesic and antiseptic properties and occasionally used internally in the treatment of pertussis, and engathol applied locally in psoriasis. The pigmentation resulting from the internal use of nitrate of silver (argyria so-called) is seldom seen in the present day, but, when epilepsy and dysentery were treated with that drug, it was not uncommon. Picric acid has been known to produce a remarkable discoloration of the skin when given internally for malaria, but it is now almost entirely used as a local application only. Sinapisms and vesicants produce brownish pigmentations on the site of application, and artificial heat, when long continued, especially on the legs, is followed by a remarkable ring-like erythema and pigmentation (erythema ab igne). Poultices may produce comedones, papulo-vesicular, and even pustular eruptions. Chemical agents used in the process of tattooing, if extensive, may led to a troublesome dermatitis from absorption. The intestinal antiseptic, salol, is capable of producing a violent general dermatitis in certain persons, and I have seen the resin of guaiacum prescribed for gout, followed by a papulo-erythematous eruption in a male patient, very similar to that which copaiba and cubebs are known to produce.

### Self-inflicted Cutaneous Lesions.

When from time to time cutaneous eruptions are seen to follow the exhibition of such simple and frequently administered drugs as borax, chlorate of potash, dulcamara, rhubarb, and many others, we are compelled more and more to study that personal peculiarity of constitution or temperament known as idiosyncrasy, to which I have previously alluded. If time permitted I should like to have dwelt upon that interesting class of self-inflicted cutaneous lesions resulting from the use of various chemical, mechanical, and therapeutic agents (Dermatitis artefacta). In investigating such cases I need only remind you here how much tact and circumspection are needed to avoid suspicion on the part of the patient that she (for they are met with almost invariably in the female sex) has been discovered in her crafty attempt to deceive. To this end, the objective evidence having led you to the conclusion that artificial means have been employed, endeavour if possible to learn the *motive* which may have prompted the deception. Inquire into the circumstances of the patient, social, socio-ethical, and domestic, for by this means valuable confirmatory evidence may often be obtained. In illustration of the above the following cases are instructive: A girl was sent to me at hospital with a severe inflammatory disorder of her feet, attributed to "broken" chilblains. The condition was clearly the result of some self-applied irritant. Upon inquiry I learnt that she was in domestic service, but complained of the number of stairs, and attributed the chief part of her ailment to the frequency with which she was required to carry heavy articles to the upper rooms. Her *motive* was to avoid stair-climbing. Another domestic servant, having been discharged from her situation, produced an acute dermatitis of the hands, in order, if possible, to be admitted into hospital, her scheme being to escape the ill-treatment to which she alleged she was subjected by her stepmother when she returned home.

I remember another instance of the same class, in which the patient, by the free application of carbolic acid, attempted so to disable herself from domestic duty that she might be allowed to return to her home in order to spend Christmas with her relatives.

Not long ago I investigated the case of a single woman for an extensive inflammatory disorder of her skin, including the face, evidently of artificial production. She admitted that her circumstances were much changed owing to disagreement with her paramour, with whom she had cohabited. Her *motive* clearly was that of extortion, to succeed in which she was anxious to secure, if possible, an opinion and statement that she had been infected with specific disease.

Another interesting case was that of a young woman who had a series of inflammatory lesions upon various parts of her body, apparently caused by a vesicant. She was admitted into hospital and closely watched, but in spite of observation others developed. Eventually she confessed to the deception when confronted by the house physician, who challenged her, he having produced a similar lesion upon his own skin by friction with a wetted finger.

Although quite apart from self-inflicted injuries I will relate one more case of clinical interest. A child was brought to me with an extensive slough over the precordia, which had existed for a fortnight. The parents were ignorant of the cause, but they informed me that the boy had recently undergone a slight operation under an anæsthetic. Upon inquiry from the anæthetist I learnt that during a period of threatened syncope twenty minims of undiluted brandy had been injected subcutaneously. In the museum of this institution there is a considerable collection of plates and coloured drawings of drug eruptions which illustrate certain groups of cases to which I have referred, and which are worthy of careful study. As I have exhausted my allotted time I will conclude by expressing the hope that all those whom I have the pleasure of addressing will, so far as opportunity allows, assist in the study and elucidation of the very important subject of drug eruptions, which to me personally is one of deep and increasing interest.

